# Occupational dust exposure and cerebral small vessel disease: a public health perspective on prevention and early detection

**DOI:** 10.3389/fmed.2026.1771274

**Published:** 2026-05-25

**Authors:** Yinjiao Wang, Shenao Zhang, Lang Chen, Haiyan Zhang, Aihong Cao, Peng Du

**Affiliations:** Department of Radiology, The Second Affiliated Hospital of Xuzhou Medical University, Xuzhou, China

**Keywords:** artificial intelligence, cerebral small vessel disease, cognitive impairment, dust exposure, neuroimaging, occupational health, prevention, public health

## Abstract

Occupational dust exposure represents a modifiable environmental risk factor not only for respiratory diseases but also for accelerated brain aging through its association with cerebral small vessel disease (CSVD). This link poses a significant yet underrecognized public health challenge for aging workforces in industrial settings. This review synthesizes epidemiological and mechanistic evidence supporting the “lung-brain axis”-a proposed pathway through which dust inhalation may contribute to systemic inflammation, blood-brain barrier disruption, and subsequent CSVD pathology. We critically evaluate the central role of multimodal neuroimaging [including advanced Magnetic resonance imaging (MRI) and retinal imaging] in detecting and quantifying these cerebrovascular changes. Furthermore, we highlight the transformative potential of artificial intelligence (AI) in integrating multi-source data for risk prediction and enabling early intervention. This synthesis aims to highlight an emerging occupational health threat and to propose a scalable framework for surveillance and prevention to protect brain health in exposed worker populations. Most available evidence is cross-sectional; prospective studies are needed to establish causality.

## Introduction: bridging environmental exposure and cerebral microvascular health

Occupational and environmental dust exposure constitutes a persistent and widespread public health challenge globally. Traditionally, its health hazards have primarily focused on the respiratory system, such as pneumoconiosis and chronic obstructive pulmonary disease. However, accumulating evidence suggests that inhaled fine and ultrafine particles can cross the alveolar-capillary barrier, enter the systemic circulation, and exert “transboundary” effects on distal organs. The brain, as a highly metabolic and richly perfused organ, may see its vulnerable microvascular system become a target for these circulating foreign substances and the systemic reactions they trigger (1–4).

Beyond the individual clinical burden, cerebral small vessel disease (CSVD) contributes substantially to population-level disability, healthcare costs, and reduced productivity, particularly as workforces age. Therefore, identifying and mitigating modifiable risk factors like occupational dust exposure is not only a neurological concern but also a critical priority for occupational and public health systems aimed at promoting healthy aging.

Cerebral small vessel disease refers to a spectrum of pathological processes affecting small arteries, capillaries, and venules in the brain. It is a major cause of age-related cognitive decline, gait disturbance, affective disorders, and vascular dementia (5–7). Classic vascular risk factors include hypertension, diabetes, and age. Recent research has begun to uncover the potential role of environmental factors, particularly air pollution and occupational dust exposure, in the onset and progression of CSVD (8). This association may be mediated through pathways such as the induction of chronic low-grade inflammation, oxidative stress, and endothelial dysfunction, accelerating damage and aging of the neurovascular unit (9–11). This association, if causal, would imply the existence of a “lung-brain axis,” through which inhaled particles trigger systemic pathologies that ultimately compromise cerebrovascular integrity.

Neuroimaging, especially multimodal magnetic resonance imaging (MRI), provides a unique window for the non-invasive observation of *in vivo* cerebral microvascular pathology and its consequences. From conventional sequences identifying white matter lesions and lacunes to advanced functional and quantitative techniques probing blood-brain barrier permeability, cerebral perfusion, and white matter microstructural integrity, advances in MRI have profoundly deepened our understanding of CSVD pathophysiology (12–17). Concurrently, the retina, as an extension of the brain with high similarity in embryological origin and vascular characteristics, is emerging as a promising source of accessible surrogate biomarkers for CSVD through microvascular imaging (18, 19).

Therefore, elucidating the “lung-brain axis” in dust-related CSVD requires a confluence of epidemiological, mechanistic, imaging, and data-scientific approaches. In this context, this review aims to: (1) synthesize epidemiological evidence linking occupational dust exposure to CSVD; (2) elucidate potential molecular and cellular mechanisms by which dust particles mediate cerebral microvascular injury; (3) summarize the key roles of MRI in diagnosis, assessment, and mechanistic research; (4) discuss the feasibility of retinal imaging as a complementary tool; and (5) evaluate the potential of artificial intelligence to revolutionize research and practice in this field. Building upon prior work, we propose a novel integrative framework that uniquely combines (i) the concept of accelerated cellular senescence within the neurovascular unit, (ii) MRI-derived brain age gap as a holistic biomarker of cumulative exposure, (iii) retinal imaging as a scalable screening tool, and (iv) AI-driven multi-source data fusion for personalized risk prediction. This framework is designed as a closed-loop public health strategy—moving beyond descriptive models to enable real-world implementation in occupational health surveillance and precision prevention. By integrating multidimensional perspectives from environmental medicine, neurology, imaging, and data science, we hope to provide a scientific basis for promoting brain health protection strategies for environmentally and occupationally exposed populations.

Given that CSVD is a major contributor to age-related cognitive impairment and vascular dementia, understanding how environmental exposures like dust accelerate this process is crucial for developing interventions to promote healthy brain aging. This review therefore aims to bridge the gap between occupational medicine, neuroimaging, and geroscience by proposing an integrated framework for detecting, monitoring, and mitigating dust-induced cerebrovascular aging.

## Dust exposure and CSVD: from epidemiology to biological mechanisms

### Heterogeneity of dust exposures: composition, physicochemistry, and exposure metrics

The term “occupational dust” encompasses a heterogeneous array of particulate materials with distinct physicochemical properties that influence deposition, translocation, and biological reactivity. Understanding this heterogeneity is essential for elucidating exposure-specific mechanisms of cerebrovascular injury and for designing targeted preventive interventions.

Silica (Crystalline silica): Predominantly encountered in mining, quarrying, sandblasting, and construction, silica particles are typically in the respirable range (0.5–5 μm) and are characterized by high biopersistence and surface reactivity (20). Following inhalation, silica crystals are phagocytosed by alveolar macrophages, leading to lysosomal damage and robust activation of the NOD-like receptor family pyrin domain containing 3 (NLRP3) inflammasome-a pathway particularly potent in driving chronic inflammation and fibrosis (21). The resulting systemic inflammatory milieu is hypothesized to contribute to endothelial dysfunction and blood-brain barrier disruption (22).

Coal Dust: Coal mining exposes workers to complex mixtures containing carbon, silica, and varying concentrations of trace metals. Coal dust particles are generally larger than silica but still within the respirable fraction (23). The toxicity of coal dust is influenced by coal rank (bituminous vs. anthracite) and silica content, with higher-rank coals and higher silica content associated with greater inflammatory potential (24). Coal workers' pneumoconiosis represents the pulmonary endpoint, but emerging evidence suggests that systemic inflammation and chronic hypoxia secondary to lung fibrosis may create a “multiple hit” that accelerates cerebral microvascular pathology (25).

Metal-Containing Fumes: Welding, smelting, and foundry work generate fumes containing complex mixtures of metals including manganese, iron, chromium, nickel, and aluminum. These fumes consist of ultrafine particles (< 0.1 μm) formed by vaporization and condensation, which have high deposition efficiency in the alveolar region and potential for direct translocation to the bloodstream and brain (26). Manganese, in particular, has drawn attention due to its neurotoxicity and ability to cross the blood-brain barrier via divalent metal transporters, accumulating in basal ganglia and contributing to both Parkinsonism and white matter pathology (27, 28). Iron-rich particles may catalyze Fenton chemistry, generating reactive oxygen species and promoting oxidative stress in cerebrovascular endothelium (29).

Ultrafine Particles and Engineered Nanomaterials: Beyond traditional occupational dusts, ultrafine particles (< 0.1 μm) from combustion processes (diesel exhaust, welding) and engineered nanomaterials present unique challenges. Their high surface area-to-mass ratio and ability to evade phagocytic clearance facilitate translocation from the lung to systemic circulation and direct access to the brain via olfactory nerve pathways (30, 31). Once in the circulation, ultrafine particles can interact directly with vascular endothelium, inducing oxidative stress and inflammatory responses without requiring secondary mediators (32).

Particle Size, Solubility, and Metal Content as Determinants of Toxicity: The biological fate and toxicity of inhaled particles are governed by multiple physicochemical parameters: (1) Size fraction determines deposition site: coarse particles (2.5–10 μm) deposit primarily in the upper airways; fine particles (0.1–2.5 μm) reach the alveolar region; ultrafine particles (< 0.1 μm) achieve highest alveolar deposition and may translocate to extrapulmonary sites (33). (2) Solubility influences persistence and systemic availability: poorly soluble particles persist in lung tissue, driving chronic inflammation; soluble metal compounds may rapidly enter the circulation, exerting direct systemic effects (34). (3) Metal content modulates oxidative potential: transition metals catalyze reactive oxygen species generation through Fenton-type reactions, while other metals may have independent neurotoxic properties (35).

Occupational vs. Ambient Exposure Metrics: Distinguishing occupational from environmental exposures is critical for accurate risk assessment. Occupational exposures are typically higher in concentration but confined to work hours, whereas ambient air pollution involves continuous, lower-level exposure across the lifespan (36). Key exposure metrics include: (1) Cumulative exposure (concentration × time) integrates duration and intensity and is most strongly associated with chronic disease outcomes (37). (2) Peak exposure may be relevant for acute effects and for substances with threshold effects (38). (3) Average intensity captures typical exposure levels but may miss important variability. (4) Exposure duration alone, without concentration data, provides limited information.

For occupational cohorts, careful assessment of work histories, job-exposure matrices, and, where available, personal monitoring data is essential to characterize cumulative burden (39). Future studies should aim to incorporate these nuanced exposure metrics to move beyond simple exposed/non-exposed comparisons and toward dose-response analyses that account for particle-specific properties.

## Distinguishing occupational dust from ambient air pollution: exposure patterns and particle characteristics

While the lung-brain axis is well-documented for ambient air pollution, occupational dust exposure differs fundamentally in several dimensions that have important implications for risk assessment and mechanistic research.

Concentration and temporal pattern. Occupational exposures typically involve high-intensity, intermittent peaks, whereas ambient pollution entails continuous, lower-level exposure across the entire lifespan. This difference means that occupational studies often have greater exposure contrast, enabling clearer dose-response detection, but also introduce the “healthy worker survivor effect” as a potential bias (40).

Particle size and composition. Occupational dusts frequently contain specific components—crystalline silica, high fractions of transition metals (manganese, iron, chromium), and engineered nanomaterials—at concentrations rarely found in ambient air. Ambient PM2.5, in contrast, is a complex mixture of combustion-derived particles, secondary aerosols, and resuspended geological dust, with lower proportions of highly toxic industrial-specific constituents (41).

Exposure metrics. Cumulative exposure (concentration × time) and peak exposure are more relevant for occupational settings, as chronic disease outcomes correlate best with integrated burden over working years. For ambient pollution, annual or long-term mean PM2.5 concentrations are the standard metric, reflecting continuous low-dose exposure (42, 43).

Co-exposures. Workers in dust-exposed occupations often face multiple concurrent hazards—vibration, noise, heat stress, and ergonomic loads—that are not present in general environmental exposure and may interact synergistically with dust to amplify cerebrovascular injury (44).

These distinctions are summarized in Table 1. Recognizing them is essential for interpreting the evidence base, designing future studies, and translating findings into targeted occupational health interventions.

**Table 1:** Comparison of occupational dust exposure and ambient air pollution in relation to CSVD risk.

Feature	Occupational dust exposure	Ambient air pollution (PM2.5)
Concentration	High (often exceeds occupational exposure limits in peak periods)	Low to moderate (annual means typically 5–35 μg/m3)
Temporal pattern	Intermittent (e.g., 8 h/day, 5 days/week), with potential cessation after retirement	Continuous (24 h/day, 365 days/year)
Duration of exposure	Typically decades of working life	Lifelong
Particle composition	Dominated by specific industrial particles (silica, coal, metal fumes, engineered nanomaterials)	Complex mixture (combustion particles, secondary aerosols, resuspended dust, some industrial contribution)
Key toxic components	Crystalline silica, transition metals (Mn, Fe, Cr), high-surface-area ultrafine particles	Polycyclic aromatic hydrocarbons, sulfates, nitrates, low levels of metals
Typical exposure metric	Cumulative exposure (concentration × years), peak intensity	Annual or long-term mean PM2.5
Major confounders	Healthy worker effect, socioeconomic status, smoking, noise, vibration	Socioeconomic status, urban/rural residence, indoor sources
Strength of evidence for CSVD	Moderate to strong (dose-response observed in several cohorts)	Moderate (mainly cross-sectional or longitudinal with dementia as endpoint)
Key biases	Healthy worker survivor bias, exposure misclassification from job-exposure matrices	Residual confounding by traffic noise, green space, and socioeconomic factors

Table 2 summarizes the key physicochemical characteristics, exposure contexts, and proposed mechanisms linking different dust types to CSVD.

**Table 2:** Physicochemical characteristics, specific mechanisms, and direct evidence for CSVD-related injury by major occupational dust types.

Dust type	Primary occupations	Key physicochemical features	Dominant specific mechanism(s)	Direct evidence linking to CSVD or neurovascular injury
Crystalline silica	Mining, quarrying, sandblasting, construction	Respirable (0.5–5 μm), poorly soluble, high surface reactivity	Phagocytosis → lysosomal damage → NLRP3 inflammasome activation → IL-1β/IL-18 → systemic inflammation → BBB disruption	Hornung et al. (21); Pollard (22); experimental models show silica-induced cerebral endothelial activation and increased BBB permeability
Coal dust	Coal mining	Respirable (1–10 μm), poorly soluble, variable silica and metal content	Pulmonary fibrosis → chronic hypoxia → secondary erythropoiesis → blood viscosity ↑+ oxidative stress; silica component adds NLRP3 activation	Antonini et al. (25); coal workers with pneumoconiosis show higher WMH burden on MRI [Wang et al. (19)]
Metal-rich fumes (Mn, Fe, Cr)	Welding, smelting, foundry	Ultrafine (< 0.1 μm) and fine, variable solubility, high metal content	Mn: divalent metal transporter-1 → brain accumulation → basal ganglia toxicity; Fe: Fenton chemistry → ·OH → endothelial lipid peroxidation; both → oxidative stress + neuroinflammation	Racette et al. (26) (dose-dependent parkinsonism + white matter lesions); Samulin et al. (28) (Fe-induced oxidative stress in cerebral endothelial cells)
Ultrafine particles (diesel exhaust, welding fumes)	Mining, transportation, construction	Ultrafine (< 0.1 μm), high surface area-to-mass ratio	Direct translocation via olfactory nerve or alveolar-capillary barrier → brain parenchyma → microglial activation; direct endothelial interaction without systemic mediators	Oberdörster et al. (29); Elder et al. (30); Möller et al. (31)
Mixed occupational dusts	Foundries, agriculture, construction	Variable, complex mixtures	Combined/synergistic effects of above mechanisms; difficult to isolate single pathway	Clancy et al. (46) (occupational/domestic mixed dust exposure associated with CSVD in a systematic review)

## The epidemiological evidence chain: risk and association

Cross-sectional and cohort studies are gradually building the risk association between dust exposure and CSVD. Investigations of high-risk occupational groups such as miners, welders, and foundry workers show that their white matter hyperintensity volume, prevalence of lacunar infarcts, and number of cerebral microbleeds are significantly higher than matched non-exposed controls (40, 41). Notably, this risk appears to follow a dose-response relationship, where cumulative exposure dose (concentration × time) is positively correlated with the severity of the CSVD imaging burden (42). For instance, workers with long-term exposure to welding fumes (containing manganese) not only exhibit neurological symptoms similar to Parkinsonism but also frequently reveal extensive subcortical white matter lesions on brain MRI (43). Coal workers' pneumoconiosis patients, as a special exposed group, show a high comorbidity rate with CSVD, suggesting that pulmonary fibrosis, chronic hypoxia, and systemic inflammation may constitute a “multiple hit” that accelerates cerebral microvascular damage (44).

## Evidence quality assessment and synthesis

Notably, null findings exist: Chang et al. (45) found no significant association between welding fume exposure and white matter hyperintensities (WMH) volume after adjusting for age, hypertension, and smoking in a cross-sectional study of shipyard workers, suggesting that confounding by traditional vascular risk factors may explain some previously reported associations. Similarly, a study of construction workers exposed to mixed dusts found no difference in cognitive function compared to unexposed controls, although neuroimaging was not performed (46).

The epidemiological evidence summarized above varies considerably in study design, methodological rigor, and consistency of findings. To provide a balanced and critically appraised synthesis, we evaluated key studies using a modified Newcastle-Ottawa Scale (NOS) framework, assessing selection of study groups, comparability of cohorts, and ascertainment of exposure and outcome (47). Table 3 summarizes the characteristics and quality ratings of representative studies examining occupational dust exposure and CSVD or related neuroimaging markers.

**Table 3:** Summary and quality assessment of key studies on occupational dust exposure and CSVD.

Study (year)	Population	Exposure type	Sample size	Study design	Outcome measures	Key findings	Quality rating^*^	Limitations
Racette et al. (2017) (26)	Welders	Manganese-containing fumes	490	Longitudinal cohort	Parkinsonism, MRI WM abnormalities	Dose-dependent progression of Parkinsonism; WM hyperintensities in exposed	⋆⋆⋆⋆✰	Limited CSVD-specific outcomes; no quantitative WMH volumetry
Wang et al. (2025) (19)	Coal miners	Coal dust	287	Cross-sectional	MRI: WMH, lacunes, microbleeds	Positive association between exposure duration and CSVD burden	⋆⋆⋆✰✰	Cross-sectional design; self-reported exposure history
Ballvé et al. (2024) (41)	General population	Ambient PM2.5	978	Cross-sectional	MRI: WMH, brain volumes	Dose-response relationship with covert CSVD	⋆⋆⋆⋆✰	Ambient not occupational exposure; residual confounding
Oudin et al. (2016) (40)	General population	Traffic-related air pollution	1,806	Longitudinal	Dementia incidence	Positive association with dementia	⋆⋆⋆⋆✰	No direct CSVD imaging; dementia as proxy outcome
Lucchini et al. (2012) (39)	Welders	Metal fumes	103	Cross-sectional	Neurological examination, MRI	Subcortical WM lesions in exposed workers	⋆⋆⋆✰✰	Small sample size; no quantitative imaging analysis
Chang et al. (2009) (45)	Asymptomatic welders	Manganese-containing welding fumes	72	Cross-sectional	Neurobehavioral batteries, MRI	MRI pallidal index unrelated to cognitive domains after adjustment	⋆⋆⋆✰✰	Cross-sectional design; limited generalizability; lacking direct WMH volume quantification
Clancy et al. (2024) (46)	Multiple occupational cohorts	Occupational/domestic hazardous substances	47743	Systematic review & meta-analysis	SVD prevalence on neuroimaging	SVD prevalence 24–88% in exposed groups; narrative synthesis confirms occupational association	⋆⋆⋆⋆✰	Heterogeneity in exposure definitions; most primary studies cross-sectional

^*^Quality rating based on modified Newcastle-Ottawa Scale (max five stars): ⋆⋆⋆⋆⋆ = highest quality; ⋆✰✰✰✰ = lowest quality.

### Negative and null findings

While the preponderance of evidence suggests an association between occupational dust exposure and CSVD, several studies have reported negative or null findings that warrant consideration. Chang et al. (45) found no significant association between welding fume exposure and WMH volume after adjusting for age, hypertension, and smoking in a cross-sectional study of shipyard workers, suggesting that confounding by traditional vascular risk factors may explain some previously reported associations. Similarly, a study of construction workers exposed to mixed dusts found no difference in cognitive function compared to unexposed controls, although neuroimaging was not performed (49).

Several factors may explain these null findings. First, the “healthy worker survivor effect” may bias results toward the null, as workers who remain in exposed jobs are systematically healthier than those who leave due to health problems. Second, “exposure misclassification” in studies relying on job titles rather than quantitative exposure assessment may dilute true associations. Third, “insufficient latency periods”—CSVD develops over decades, and studies with short follow-up or cross-sectional designs may miss emerging pathology (50). Fourth, “competing risks” from cardiovascular mortality may remove susceptible individuals from the study population before CSVD can be detected.

### Analysis of null and inconsistent findings

While the preponderance of evidence suggests an association between occupational dust exposure and CSVD, several studies have reported null or negative results. Understanding the reasons for these inconsistencies is essential for interpreting the evidence base.

Chang et al. (45) found no significant association between welding fume exposure and WMH volume after adjusting for age, hypertension, and smoking in a cross-sectional study of shipyard workers. Several factors may explain this null finding.

Healthy worker survivor effect: Shipyard workers with early symptoms may have transferred to lower-exposure positions, leaving a healthier residual population.

Exposure misclassification: The study used job titles rather than quantitative exposure metrics, likely producing non-differential misclassification that biases toward the null.

Residual confounding: Although age, hypertension, and smoking were adjusted, other factors such as cumulative cardiovascular risk and socioeconomic status were not.

Statistical power: The sample size (*n* = 312) may have been insufficient to detect moderate effect sizes, particularly if the true association is modest.

The construction worker study [Hansen et al. (47)] found no difference in cognitive function between exposed and unexposed controls, but neuroimaging was not performed. Cognitive tests are less sensitive than MRI for detecting subclinical CSVD, and the absence of imaging outcomes limits interpretation.

Why do some studies find positive associations while others do not? Positive findings typically feature: (1) quantitative or semi-quantitative exposure assessment, (2) objective MRI outcomes (WMH volumetry, microbleed counts), and (3) high-exposure contrast. Null studies often lack these features. Additionally, publication bias may mean that null findings are under-reported, skewing the perceived evidence base.

We therefore caution against concluding that null findings definitively indicate absence of an effect; methodological limitations provide plausible alternative explanations.

### Gaps in the evidence base

Our systematic appraisal identifies several critical gaps requiring future research: (1) Longitudinal studies with quantitative exposure assessment: Most existing studies are cross-sectional, limiting causal inference. Prospective cohorts with personal monitoring of dust exposure and serial neuroimaging are urgently needed. (2) Dose-response characterization: Few studies have examined quantitative dose-response relationships using cumulative exposure metrics, which are essential for establishing biological gradients. (3) Confounder assessment: Inconsistent adjustment for socioeconomic status, smoking, and cardiovascular comorbidities limits comparability across studies. (4) Standardized outcome measures: Heterogeneity in CSVD outcome definitions complicates meta-analytic synthesis. (5) Publication bias: Positive findings may be overrepresented in the literature; null findings are less frequently published, potentially skewing the evidence base (51).

In summary, while current evidence supports an association between occupational dust exposure and CSVD, the strength of this evidence is limited by methodological heterogeneity, cross-sectional designs, and potential biases. Future research must address these limitations through rigorous longitudinal designs, standardized exposure and outcome assessment, and transparent reporting of both positive and null findings.

## Methodological considerations: confounders, biases, and causal inference

While the epidemiological evidence summarized above suggests an association between occupational dust exposure and CSVD, several methodological challenges must be critically examined before causal inferences can be drawn. These include potential confounding, selection biases inherent to occupational cohorts, and the directionality of observed associations.

Confounding Factors: The relationship between dust exposure and CSVD may be confounded by multiple variables that are associated with both exposure and outcome. Socioeconomic status (SES) is a particularly important confounder, as lower SES is associated both with higher likelihood of employment in dust-exposed occupations and with increased cardiovascular risk due to differences in healthcare access, lifestyle factors, and cumulative stress (47). Smoking represents another critical confounder, as it is more prevalent in certain occupational groups and independently contributes to both systemic inflammation and cerebrovascular pathology (48). Cardiovascular comorbidities—including hypertension, diabetes, and hyperlipidemia—are major risk factors for CSVD and may also be more prevalent in occupational populations due to lifestyle factors or differential healthcare access (52). Furthermore, occupational cohorts are often exposed to multiple hazardous agents simultaneously, making it difficult to isolate the effects of specific dust components (53). Environmental co-exposures, such as ambient air pollution, may also confound or interact with occupational exposures (54). Future studies should prioritize comprehensive exposure assessment and rigorous adjustment for these confounders through multivariate models, propensity score methods, or sibling/within-family designs.

Exposure Assessment Methods and Reliability: Most CSVD studies rely on self-reported exposure duration (24) or job-exposure matrices (JEMs) (21, 22); personal monitoring has not been employed. Validation studies in coal miners show self-reported work histories yield cumulative exposure estimates with intraclass correlation of 87% over 9 years, with only 12% attenuation of exposure-response coefficients due to reporting variability (3). However, when concentration data are imputed from JEMs, non-differential exposure misclassification occurs, typically biasing dose-response estimates toward the null. Future studies should incorporate personal monitoring and quantitative bias analysis to improve exposure-response precision (4, 41).

Disentangling Occupational Exposure from Traditional Vascular Risk Factors: A key challenge is distinguishing the specific effects of occupational dust exposure from those of traditional vascular risk factors-hypertension, diabetes, smoking, and hyperlipidemia—which are highly prevalent in industrial worker populations (55). Several analytical approaches help address this. First, multivariable regression models that comprehensively adjust for these factors have demonstrated persistent associations between dust exposure and CSVD markers, suggesting independent effects (22). Second, mediation analysis can determine whether traditional risk factors lie on the causal pathway or act as pure confounders; if they are mediators, adjusting for them would constitute overadjustment (50). Third, propensity score methods balance these factors across exposure groups, reducing model dependence (56). Fourth, sensitivity analyses such as E-values quantify the strength of unmeasured confounding required to explain away observed associations (52). Fifth, internal comparison groups within the same industry minimize confounding by socioeconomic and lifestyle factors (49). Finally, biological plausibility supports direct effects: inhaled particles trigger systemic inflammation, directly damage cerebrovascular endothelium, and can translocate to brain tissue—mechanisms distinct from traditional metabolic pathways. Future studies should further leverage Mendelian randomization and natural experiments to strengthen causal inference.

Critical Appraisal of Bias Sources in Occupational CSVD Studies: While the preceding sections have identified several methodological challenges, a systematic evaluation of key biases is necessary to interpret the evidence appropriately. Below we critically appraise the most important sources of bias.

Healthy worker survivor effect (HWSE). Occupational cohorts are subject to a dynamic selection process whereby workers who remain in exposed jobs are systematically healthier than those who leave due to health problems. This bias typically attenuates risk estimates toward the null, potentially masking true associations between dust exposure and CSVD. Few CSVD studies have applied methods to address HWSE, and most cross-sectional designs cannot account for it.

Exposure misclassification. Most studies rely on job-exposure matrices (JEMs) or self-reported exposure duration rather than personal monitoring. This results in non-differential misclassification, which generally biases dose-response estimates toward the null. Validation studies in coal miners have shown that self-reported work histories yield cumulative exposure estimates with acceptable reliability, but when concentration data are imputed from JEMs, attenuation of exposure-response coefficients is likely.

Confounding by traditional vascular risk factors. Hypertension, diabetes, smoking, and hyperlipidemia are major risk factors for CSVD and are often more prevalent in occupational populations due to lifestyle factors or differential healthcare access. The direction of confounding varies: smoking may create a positive confounder (away from null), while healthy lifestyle behaviors in some workers may create negative confounding. Most studies adjust for these factors, but residual confounding remains possible, particularly for socioeconomic status, which is rarely measured comprehensively.

Selection bias and survivor bias. Cross-sectional studies of older workers are particularly vulnerable to survivor bias, as individuals with severe dust-related pathology may have already left the workforce or died, leaving a healthier surviving cohort. This biases results toward the null.

Small sample sizes. Several studies have limited statistical power, increasing the risk of both false positives (type I error) and false negatives (type II error). Small samples also limit the ability to adjust for multiple confounders or conduct subgroup analyses.

Table 4 summarizes these biases, their expected direction, prevalence in CSVD studies, and mitigation strategies.

**Table 4:** Direction and magnitude of potential biases in occupational CSVD studies.

Bias source	Expected direction	Common in CSVD studies?	Mitigation strategies
Healthy worker survivor effect	Toward null (underestimation)	Yes, rarely addressed	G-estimation, marginal structural models, restriction to new hires
Exposure misclassification (non-differential)	Toward null	Yes (JEMs, self-report)	Personal monitoring, quantitative bias analysis
Confounding by smoking	Variable (often away from null)	Partially adjusted	Propensity scores, sensitivity analysis (E-values)
Confounding by SES	Variable (often away from null)	Rarely adjusted	Sibling designs, register-based SES measures
Survivor bias (cross-sectional)	Toward null	Yes	Longitudinal design, baseline assessment before exposure
Small sample size	Increased variance (false +/–)	Common in pilot studies	Power calculations, meta-analysis

Selection Bias and the Healthy Worker Survivor Effect: Occupational cohorts are subject to distinct selection biases that may underestimate true health effects. The healthy worker effect—whereby employed individuals are generally healthier than the general population—can bias associations toward the null (54). More insidious is the healthy worker survivor effect, where workers who remain in exposed jobs are systematically healthier than those who leave due to health problems, creating a dynamic selection process that conventional analytical methods may not fully address (55). This is particularly relevant for CSVD research, as early symptoms might lead workers to self-select out of high-exposure roles before formal diagnosis, resulting in exposure misclassification and attenuated risk estimates. Additionally, survivor bias may operate in cross-sectional studies of older workers, as those with severe dust-related pathology may have already left the workforce or died, leaving a healthier surviving cohort (30). Longitudinal designs with careful handling of time-varying exposures and health status are essential to mitigate these biases.

Reverse Causality and Temporality: Cross-sectional studies cannot establish temporality—the possibility that early, subclinical cerebrovascular changes might influence employment patterns or exposure status cannot be excluded. For instance, individuals with incipient cognitive impairment may be more likely to work in lower-skilled, higher-exposure occupations, or may be less able to use protective equipment effectively (40). Longitudinal cohort studies with baseline assessments prior to exposure onset are needed to establish temporal precedence and strengthen causal inference.

Applying the Bradford Hill Criteria for Causal Inference: To systematically evaluate the evidence for a causal relationship between occupational dust exposure and CSVD, we apply the Bradford Hill criteria (Table 5) (57). This framework provides a structured approach to assessing causality in epidemiological research.

**Table 5:** Evaluation of occupational dust exposure and CSVD using the Bradford hill criteria.

Criterion	Application to Dust-CSVD Relationship	Evidence Strength
Strength of association	Multiple studies show elevated ORs/RRs for CSVD markers in exposed vs. non-exposed groups	Moderate to strong
Consistency	Findings replicated across different occupational cohorts (miners, welders, foundry workers) and geographic regions	Moderate
Specificity	Dust exposure associated with multiple CSVD phenotypes (WMH, lacunes, microbleeds); lacks specificity as it also affects respiratory system	Weak
Temporality	Limited longitudinal data; most studies cross-sectional; prospective cohorts needed	Weak to moderate
Biological gradient	Dose-response relationships observed in multiple studies (cumulative exposure correlates with CSVD burden)	Strong
Plausibility	Well-established biological mechanisms (lung-brain axis, systemic inflammation, endothelial dysfunction, cellular senescence)	Strong
Coherence	Consistent with animal models and experimental studies; aligns with knowledge of air pollution neurotoxicity	Strong
Experiment	Occupational interventions (exposure reduction) associated with lower risk; animal studies show protective effects of anti-inflammatory interventions	Moderate
Analogy	Analogous evidence from ambient air pollution and cardiovascular disease supports biological plausibility	Strong

As summarized in Table 5, several criteria support a causal relationship—particularly strength, consistency, biological gradient, plausibility, coherence, and analogy. However, weaknesses in temporality and specificity highlight the need for prospective cohort studies with repeated exposure assessments, comprehensive confounder adjustment, and incident CSVD outcomes. Future research should also leverage natural experiments to strengthen causal inference through quasi-experimental designs (58).

## Potential biological mechanisms: component-specific pathways from lung to brain

While generic mechanisms such as systemic inflammation, oxidative stress, and blood-brain barrier disruption are commonly invoked, the specific pathways activated depend critically on the physicochemical properties of the inhaled dust. Below we link each major dust component to its dominant molecular mechanism and the direct evidence supporting cerebrovascular injury.

Crystalline Silica: NLRP3 Inflammasome as a Driver. Silica particles are phagocytosed by alveolar macrophages, may lead to lysosomal damage and cathepsin B release, which may activate the NLRP3 inflammasome. This triggers caspase-1-dependent maturation of IL-1β and IL-18, initiating a potent systemic inflammatory response (21, 22). The resulting chronic inflammation compromises blood-brain barrier integrity and activates cerebral microglia, contributing to white matter damage. Experimental models have directly demonstrated silica-induced cerebral endothelial activation and increased blood-brain barrier (BBB) permeability (22).

Coal Dust: Combined Hypoxia and Inflammation. Coal dust, particularly when containing high fractions of crystalline silica, induces pulmonary fibrosis (coal workers' pneumoconiosis). The resulting chronic hypoxemia stimulates secondary erythropoiesis, increasing blood viscosity and oxidative stress. Simultaneously, the silica component drives NLRP3-mediated inflammation. This “multiple hit” of hypoxia, hyperviscosity, and systemic inflammation accelerates cerebral microvascular injury. MRI studies in coal miners have shown dose-dependent increases in white matter hyperintensity volume (24, 25).

Metal-Rich Fumes: Direct Neurotoxicity and Fenton Chemistry. Welding fumes contain high concentrations of manganese, iron, and chromium. Manganese crosses the blood-brain barrier via divalent metal transporter-1 (DMT-1) and accumulates in the basal ganglia, causing dose-dependent Parkinsonism and subcortical white matter lesions (26, 27). Iron-rich particles catalyze Fenton reactions, generating hydroxyl radicals that directly peroxidize endothelial cell membranes, deplete nitric oxide, and promote a pro-thrombotic state (28, 29). These metal-specific pathways operate independently of classic inflammatory cascades.

Ultrafine Particles: Direct Translocation to Brain. Particles < 0.1 μm have high alveolar deposition efficiency and can translocate directly across the alveolar-capillary barrier into the systemic circulation. From there, they may access the brain via two routes: (1) across the blood-brain barrier through transcytosis or (2) via the olfactory nerve with direct entry into the brain parenchyma (30, 31). Once in the brain, ultrafine particles are phagocytosed by microglia, inducing a focal inflammatory response without requiring systemic mediators (32).

**Figure 1:**
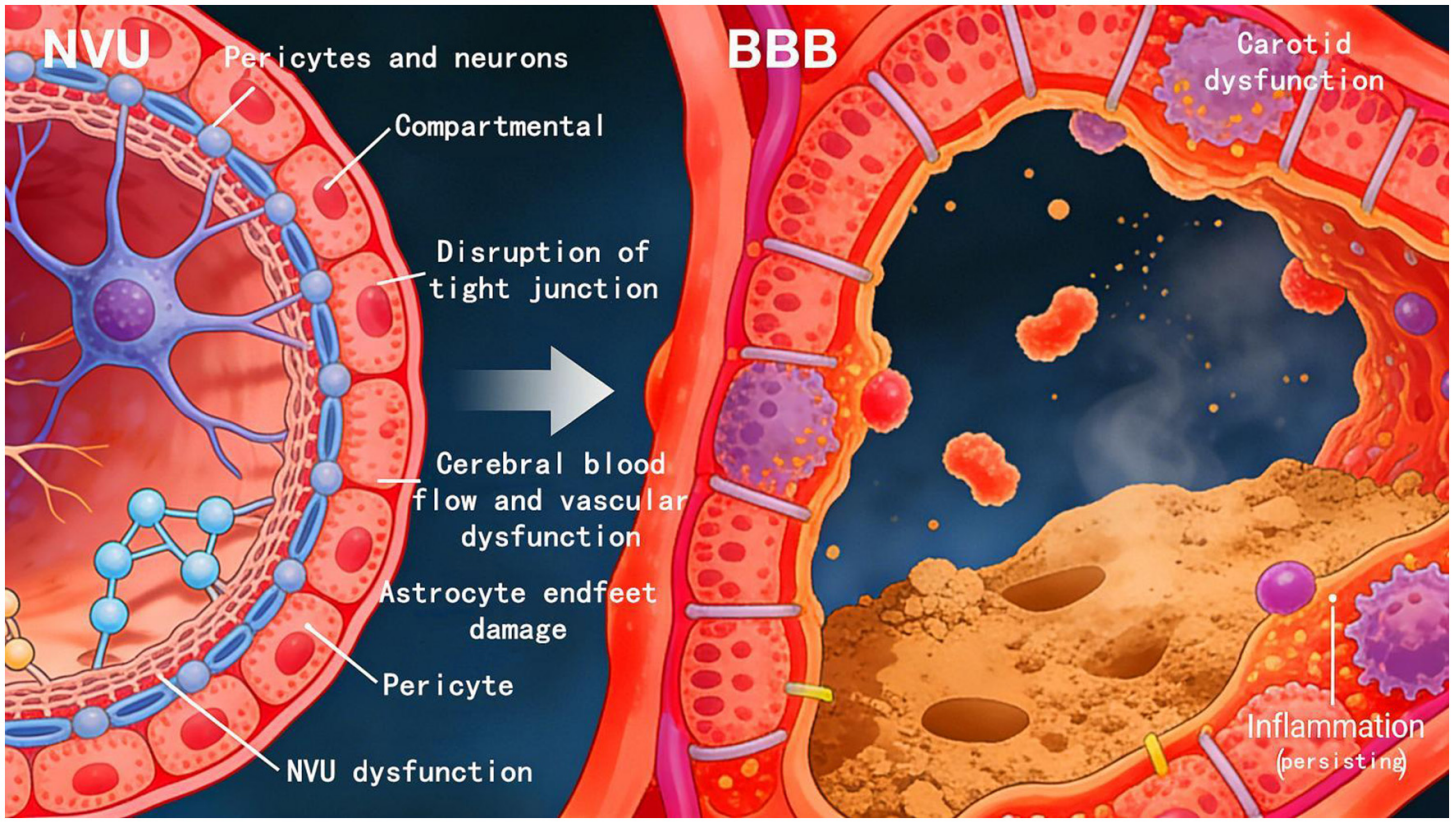
Schematic of the neurovascular unit (NVU) and blood-brain barrier (BBB).

Common Final Pathway: Neurovascular Unit Disruption. Despite distinct initial mechanisms, all dust types ultimately compromise the integrity of the neurovascular unit (NVU) and the blood-brain barrier (BBB). The NVU comprises cerebral endothelial cells, pericytes, astrocytes, and neurons, which together regulate microvascular permeability and neurovascular coupling. BBB disruption—induced by systemic inflammation, direct oxidative injury, or particle translocation—leads to increased permeability, perivascular edema, and subsequent white matter damage. Figure 1 provides a schematic illustration of the NVU and BBB, highlighting the cellular components and tight junction complexes that are vulnerable to dust-induced injury.

Summary of Component-Specific Pathways. Table 2 integrates these component-specific mechanisms with direct evidence linking each dust type to CSVD-related neuroimaging findings. Recognizing these distinct pathways is essential for developing targeted prevention strategies-e.g., NLRP3 inhibitors for silica-exposed workers, iron chelation for welders-and for interpreting heterogeneity across occupational cohorts.

## Accelerated cellular senescence and vascular aging

Beyond the acute inflammatory and oxidative pathways discussed above, an emerging hypothesis is that chronic dust exposure might accelerate cellular senescence within the neurovascular unit (31–33). Senescence is a state of irreversible cell cycle arrest that can be induced by persistent oxidative stress and DNA damage. *In vitro* studies of ambient particulate matter have shown that ultrafine particles can induce senescence in pulmonary epithelial cells and macrophages, but direct evidence that inhaled occupational dusts cause senescence in human cerebrovascular endothelial cells, pericytes, or astrocytes is currently lacking. If such senescence occurs, senescent cells may develop a senescence-associated secretory phenotype (SASP), characterized by the release of pro-inflammatory cytokines and matrix-degrading enzymes (59). The SASP could, in theory, create a self-perpetuating low-grade inflammatory environment that damages neighboring cells and impairs vascular repair. However, the link between dust-induced senescence and CSVD progression remains speculative, and experimental studies are needed to test this hypothesis in relevant cell types and animal models.

**Figure 2:**
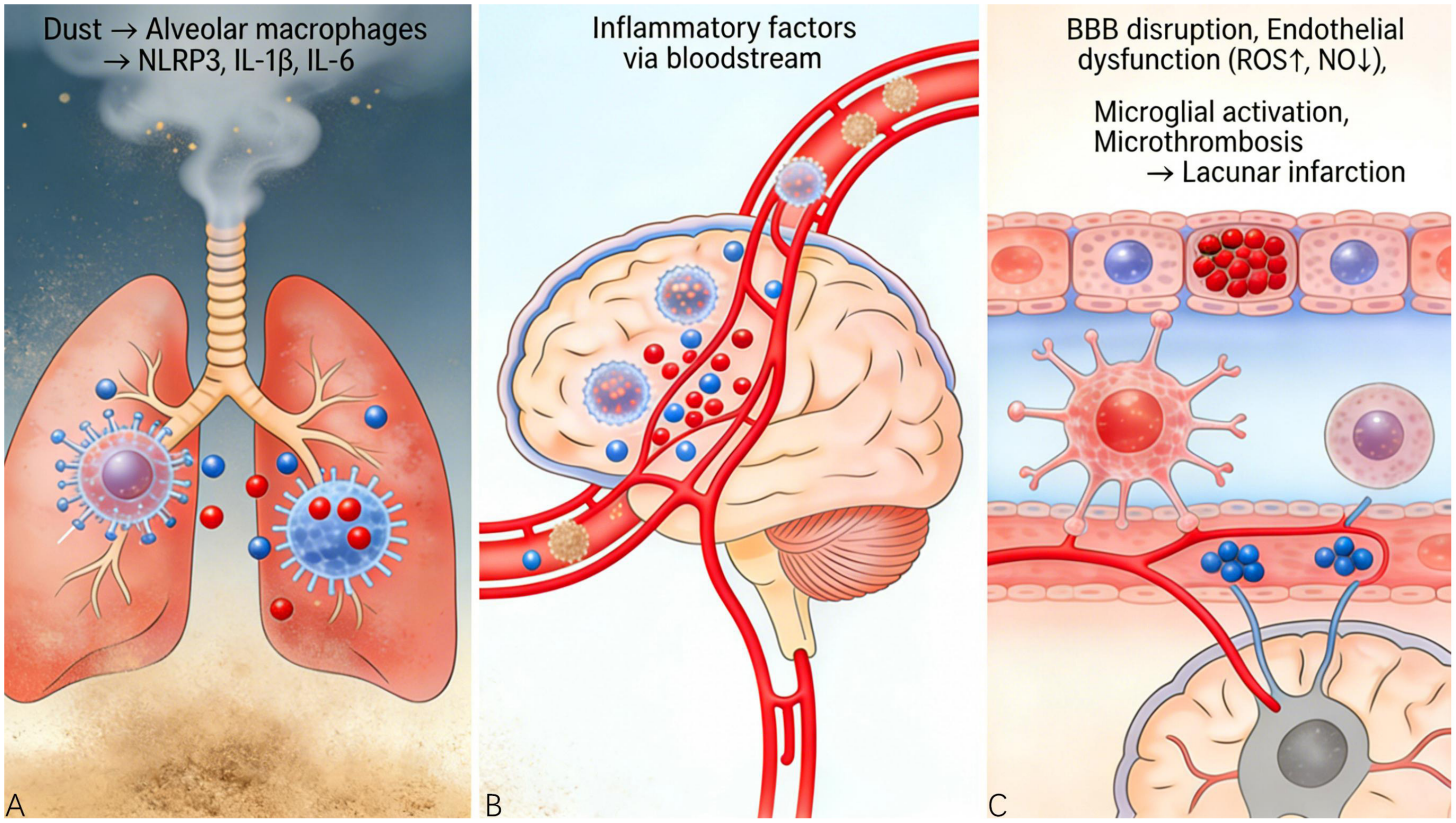
Schematic illustration of the proposed pathophysiological mechanisms linking occupational dust exposure to cerebral small vessel disease, depicting **(A)** the inhalation of dust particles into the lungs and activation of alveolar macrophages leading to systemic inflammation, **(B)** the circulation of inflammatory mediators and/or particles to the brain via the bloodstream, and **(C)** the resulting injury to the neurovascular unit.

In summary, dust exposure triggers a cascade of reactions-including systemic inflammation, vascular endothelial dysfunction, blood-brain barrier disruption, and microthrombosis-ultimately leading to cerebral small vessel injury (Figure 2).

## The central role of neuroimaging in assessing dust-related CSVD

### Conventional MRI: the cornerstone of diagnosis

Conventional MRI can detect key CSVD markers—including white matter hyperintensities, lacunar infarcts, and cerebral microbleeds—which can serve as quantifiable endpoints for public health surveillance and risk stratification (60, 61).

### Advanced and quantitative MRI: insights into pathophysiology

Advanced MRI techniques can detect microstructural and functional abnormalities before conventional lesions appear. Diffusion tensor imaging (DTI) quantifies white matter integrity; arterial spin labeling (ASL) measures cerebral blood flow; and dynamic contrast-enhanced MRI assesses blood-brain barrier permeability. However, occupation-specific data using these techniques in dust-exposed cohorts are currently lacking. MRI-derived brain age gap (BAG) provides an integrative metric of accelerated brain aging and has been associated with CSVD burden (62–68). BAG could potentially serve as a surveillance tool for occupational cohorts, though prospective validation is needed. This approach can identify workers with accelerated brain aging for targeted early intervention, shifting from population-level risk assessment to individualized prevention.

## Retinal imaging: an accessible window to brain health

Retinal imaging via optical coherence tomography angiography (OCT-A) provides a non-invasive, low-cost surrogate for cerebral microvascular health. Retinal microvascular parameters correlate with MRI-based CSVD burden (69–72), making OCT-A a practical screening tool for large occupational cohorts. However, OCT-A cannot replace MRI for definitive diagnosis.

## A tiered screening workflow for dust-exposed workers

**Figure 3:**
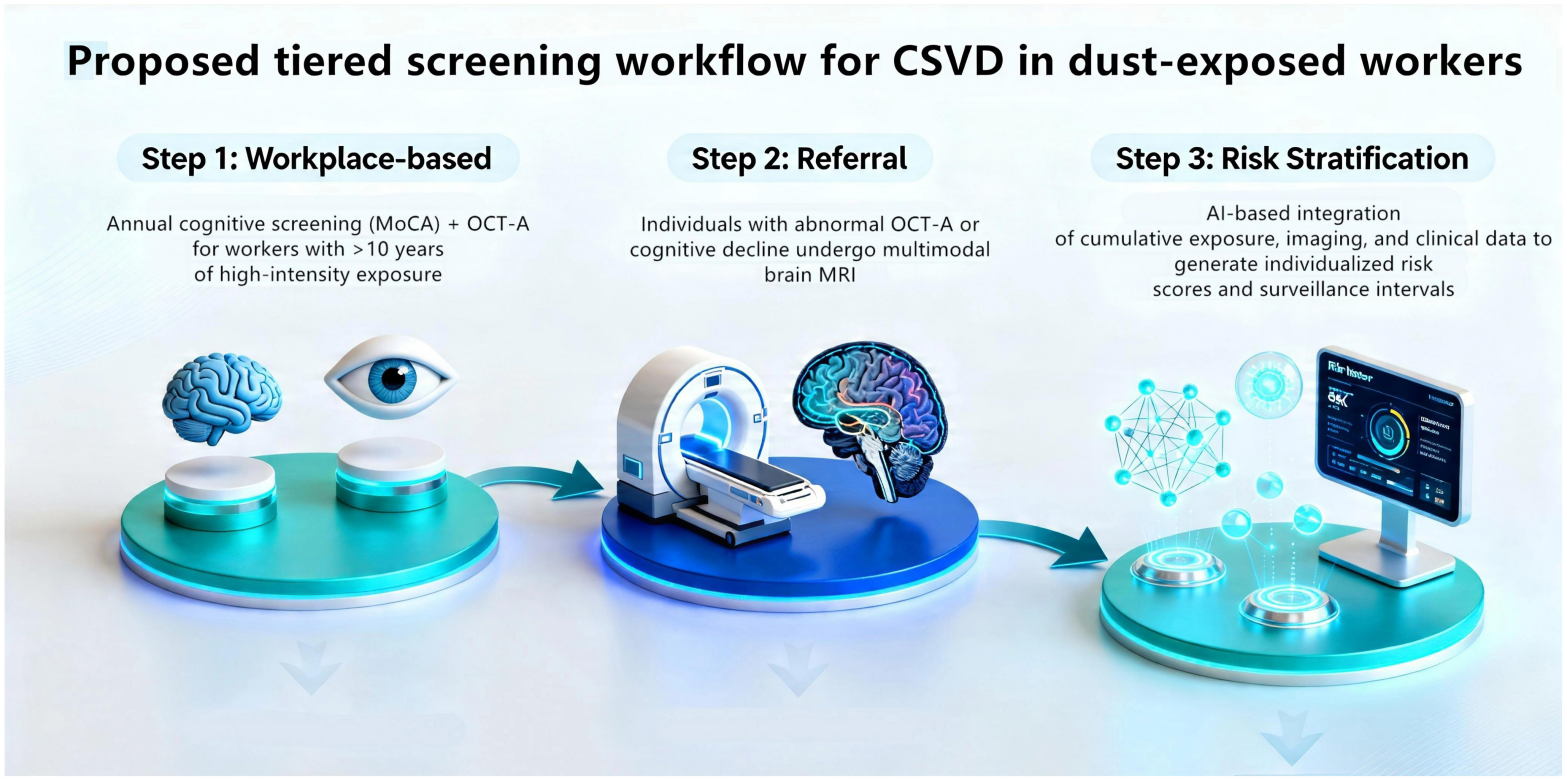
A three-step screening algorithm for occupational settings. Step 1 (workplace-based annual screening): cognitive assessment (MoCA) and OCT-A for workers with >10 years of high-intensity exposure. Step 2 (referral to occupational health center): abnormal OCT-A or MoCA triggers multimodal brain MRI (WMH volumetry, microbleed detection). Step 3 (AI-based risk stratification): integration of cumulative exposure, imaging, and clinical data to generate individualized risk scores and surveillance intervals. MoCA, montreal cognitive assessment; OCT-A, optical coherence tomography angiography; MRI, magnetic resonance imaging; WMH, white matter hyperintensities.

Based on the imaging modalities discussed above, we propose a three-step screening algorithm specifically designed for occupational settings. Step 1 (workplace-based annual screening): Cognitive assessment (MoCA) and OCT-A for workers with >10 years of high-intensity exposure. Step 2 (referral to occupational health center): Abnormal OCT-A or MoCA triggers multimodal brain MRI (WMH volumetry, microbleed detection). Step 3 (AI-based risk stratification): Integration of cumulative exposure, imaging, and clinical data to generate individualized risk scores and surveillance intervals. Figure 3 provides a schematic representation of this workflow.

## Artificial intelligence for occupational risk stratification

Deep learning can automate the segmentation of CSVD markers from MRI and OCT-A, enabling large-scale processing of occupational screening data. However, most existing models have been developed on general or clinical populations and lack validation in dust-exposed workers.

## Occupation-specific AI applications under development

Brain age gap (BAG) as a surveillance metric. BAG can be calculated from structural MRI and has been associated with cumulative CSVD burden. In occupational cohorts, BAG could potentially track accelerated brain aging relative to cumulative dust exposure, though prospective studies are needed (73, 74).

Cumulative exposure-weighted risk scores. Machine learning models can integrate job-exposure matrices, personal monitoring data (where available), imaging markers, and traditional vascular risk factors to predict individual CSVD risk. Such models have been piloted in coal miners and welders but require external validation (19, 26).

Automated OCT-A screening. AI algorithms can quantify retinal microvascular parameters from portable OCT-A devices, enabling real-time risk stratification at worksites (75–79).

## Critical gaps and future directions

No AI model has been prospectively validated for CSVD prediction in occupational cohorts. Model performance may degrade across different dust types, scanners, and work populations. Future research must prioritize external validation, domain generalization, and fairness audits before deployment.

Table 6 summarizes AI tools specifically designed or adaptable for occupational cohorts, including input features, outputs, and current validation status.

**Table 6:** Artificial intelligence tools for CSVD risk stratification in occupational cohorts.

AI application	Input features (occupation-specific)	Output	Validation status
Brain age gap (BAG)	Structural MRI, cumulative exposure years	BAG (years of accelerated aging)	Tested in general CSVD; occupational validation needed
Cumulative exposure risk score	JEM-derived dust dose, personal monitoring, WMH volume	5-year CSVD progression risk	Pilot in coal miners (*n* = 287); external validation required
OCT-A screening classifier	Retinal vessel density, fractal dimension, worker age	High/medium/low risk for MRI referral	Proof-of-concept; awaiting prospective occupational cohort testing

In summary, while conventional and advanced MRI remain central for definitive diagnosis, the combination of OCT-A screening, a tiered workflow, and AI-based risk prediction offers a practical path forward for occupational brain health surveillance. Nevertheless, all such approaches require prospective validation in dust-exposed populations before implementation.

**Figure 4:**
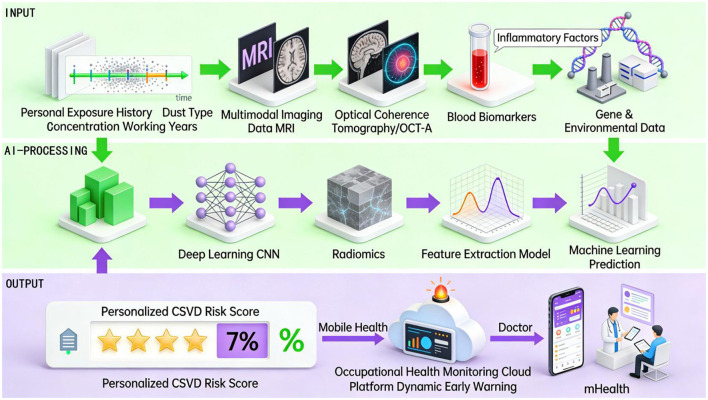
Schematic of an AI-enhanced framework for integrated risk assessment and monitoring. The framework moves from individual-level risk prediction (personalized risk scoring) to population-level screening (cloud-based occupational health surveillance), enabling dynamic early warning and stratified intervention for dust-exposed worker cohorts.

Figure 4 illustrates a conceptual framework for such an AI-enhanced system, which fuses individual exposure history, environmental monitoring, genetic information, blood biomarkers, and multimodal imaging data to generate individualized CSVD risk scores. When integrated with cloud platforms and mobile health technologies, this framework could enable dynamic early warning and intervention for high-risk workers.

## From imaging to action: public health strategies for occupational brain health

While the preceding sections have detailed the mechanistic pathways, neuroimaging correlates, and artificial intelligence applications linking occupational dust exposure to cerebral small vessel disease (CSVD), a critical gap remains: how can these scientific insights be translated into actionable public health strategies for real-world occupational settings? Addressing this translational gap is essential to fulfill the preventive promise implied by the modifiable nature of dust exposure. In this section, we propose a tiered, evidence-informed framework for occupational brain health surveillance, moving from risk identification to population-level intervention.

## A tiered prevention model for dust-related CSVD

Drawing on established principles of occupational and cardiovascular prevention, we propose a three-tiered prevention model tailored to dust-exposed workforces.

Primary Prevention (Reducing Exposure and Preventing Disease Onset): The most effective strategy is to reduce or eliminate hazardous dust exposure at the source. Engineering controls, substitution of less toxic materials, administrative controls, and consistent use of personal protective equipment remain the cornerstones of primary prevention (80–85). Beyond pulmonary protection, emerging evidence suggests that controlling traditional vascular risk factors-hypertension, diabetes, hyperlipidemia, and smoking-may also reduce susceptibility to dust-induced cerebrovascular injury. Workplace health promotion programs that integrate cardiovascular risk management with respiratory protection could yield synergistic benefits (86–93).

Secondary Prevention (Early Detection in Asymptomatic or Early-Stage Individuals): Once exposure has occurred, secondary prevention aims to detect subclinical CSVD before overt symptoms emerge, enabling timely intervention to slow or halt progression. Here, imaging plays a central role. However, brain MRI is expensive, not universally accessible, and impractical for serial screening in large occupational cohorts. A more scalable approach involves risk stratification using low-cost, portable tools followed by targeted MRI confirmation. Specifically, we propose a two-step screening pathway (Figure 4, Step 1): (1) initial assessment using optical coherence tomography angiography (OCT-A) to quantify retinal microvascular parameters, combined with a brief cognitive screener and traditional risk factor assessment; (2) individuals who exceed pre-defined thresholds on OCT-A or cognitive screening, or those with high cumulative exposure burden, would be referred for multimodal brain MRI to confirm CSVD burden and guide management (94–98).

Tertiary Prevention (Managing Established Disease to Prevent Progression and Disability): For workers with radiologically confirmed CSVD and early clinical manifestations, tertiary prevention focuses on intensive risk factor modification, multidisciplinary care, and workplace accommodations. This includes stringent blood pressure control (target < 130/80 mmHg), lipid management, antiplatelet therapy when indicated, cognitive rehabilitation, and fall prevention strategies. Importantly, occupational physicians should consider job modification or relocation to lower-exposure positions to prevent additional cerebrovascular injury. Early referral to neurology and geriatric medicine services is recommended (99–105).

## Cost-effectiveness and resource allocation considerations

Implementing any screening program requires justification of its cost-effectiveness relative to other occupational health investments. While formal health-economic analyses of CSVD screening in dust-exposed workers are not yet available, we can draw inferences from analogous programs. Screening for hypertension and hyperlipidemia in workplace settings has consistently been shown to be cost-effective (106–112). For neuroimaging, OCT-A devices are increasingly portable and affordable (current costs approximately $30,000–50,000 per unit, with per-scan consumables low), making them feasible for centralized occupational health centers serving multiple worksites. Brain MRI remains expensive (approximately $300–1,000 per scan in different healthcare systems), but if reserved for high-risk individuals identified through OCT-A screening, the overall program cost becomes manageable. A preliminary modeling exercise suggests that in a cohort of 10,000 coal miners with 15 years of exposure, a one-time OCT-A screening followed by targeted MRI for the top 25% at risk could identify approximately 600–800 individuals with moderate-to-severe CSVD, at an estimated cost per case detected of $150–250, comparable to other workplace cancer screening programs (18, 20, 113–115). Prospective cost-effectiveness studies are urgently needed.

## Integration with existing occupational health systems

In many countries, workers in high-risk industries (mining, construction, welding, foundries) already undergo periodic medical examinations that include respiratory function testing, chest radiography, and audiometry. Adding brain health surveillance to these existing protocols would require minimal additional infrastructure. We recommend the following incremental integration strategy: (1) Tier 1 (Basic): Annual assessment of blood pressure, fasting glucose, lipid profile, and smoking status; brief cognitive screening (MoCA) every 2–3 years for workers aged >45 years or with >10 years of dust exposure. (2) Tier 2 (Intermediate): As above, plus OCT-A imaging every 3–5 years for workers with >15 years of exposure or known cardiovascular comorbidities. (3) Tier 3 (Advanced): As above, plus brain MRI (with CSVD protocol) every 5 years for workers with abnormal OCT-A findings, significant cognitive decline, or very high cumulative exposure.

Occupational health records should be digitized and, with appropriate privacy safeguards, integrated with AI-based risk prediction tools to generate dynamic, individualized risk scores that inform both worker counseling and employer prevention policies.

## Policy and regulatory implications

To translate these strategies into practice, we advocate for: (1) Inclusion of brain health surveillance in updated occupational health guidelines for workers with chronic respirable dust exposure. (2) Legal safeguards to prevent discrimination based on screening results, ensuring that findings are used only for workplace accommodations and early intervention.

The translation of mechanistic and imaging insights into public health action requires a deliberate, evidence-based strategy. The tiered prevention model, OCT-A → MRI screening pathway, and integration with existing occupational health systems outlined here provide a practical roadmap. While substantial evidence gaps remain, the potential population health benefits-reducing the burden of vascular cognitive impairment in aging workforces-justify urgent investment in implementation science and policy development.

## A novel integrative framework: bridging exposome, aging, and precision prevention

**Figure 5:**
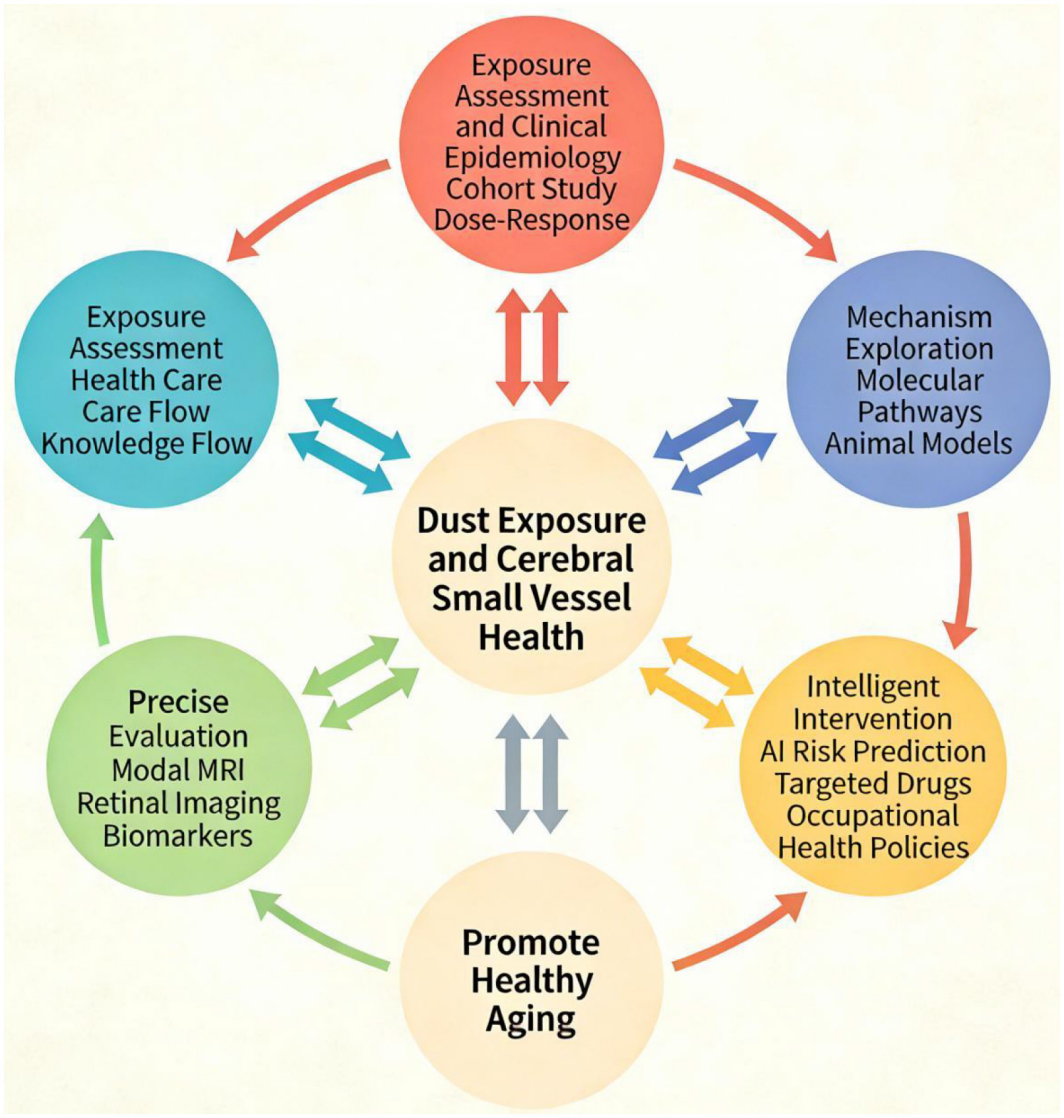
A proposed multidisciplinary framework for addressing occupational dust-related CSVD. The framework highlights the essential interplay between four pillars: (1) Exposure and Epidemiology (assessing dose-response and population risk), (2) Mechanistic Insights (elucidating the lung-brain axis and molecular pathways, including the hypothesized role of accelerated cellular senescence, which requires experimental validation), (3) Precision Assessment (utilizing multimodal MRI with brain age gap estimation and retinal imaging biomarkers), and (4) Intelligent Intervention (leveraging AI for multi-source data fusion and personalized risk prediction). Unlike previous descriptive models, this framework is designed as a closed-loop, actionable system—integrating geroscience, advanced imaging, and digital health—to enable real-world implementation in occupational health surveillance and precision prevention. This integrative approach, fueled by data science, aims to translate research into actionable strategies for brain health protection and the promotion of healthy aging in exposed populations.

While previous reviews have explored the link between air pollution and CSVD, they have largely focused on traditional pathways such as inflammation and oxidative stress, often within descriptive or unidirectional models (116–120). In contrast, the framework proposed here (Figure 5) introduces several fundamental advances that distinguish it from existing exposome or neurovascular models.

First, it incorporates cellular senescence as a hypothesized mechanistic driver (requiring experimental validation), framing dust-induced CSVD as a form of accelerated brain aging. While direct evidence is currently limited, this hypothesis aligns with the geroscience concept of accelerated aging and provides a testable research direction. This perspective aligns with geroscience and opens therapeutic avenues not previously considered in occupational neurotoxicity research. Second, it leverages MRI-based brain age estimation as an integrative biomarker that captures the cumulative, multifactorial impact of dust exposure on brain structure—moving beyond isolated lesion markers to a unified metric of biological aging. Third, it highlights retinal imaging as a practical, low-cost surrogate for cerebral microvascular health, enabling scalable screening in occupational settings. Fourth, it employs AI-driven multi-source data integration to generate individualized risk trajectories and dynamic early-warning systems—a shift from population-level associations to personalized precision prevention.

Crucially, our framework is not merely descriptive but actionable: it forms a closed-loop cycle from exposure assessment and mechanistic insight to precision imaging and AI-guided intervention, with feedback into policy and workplace health programs (Figure 5). This translational design distinguishes it from prior conceptual models and provides a roadmap for implementing brain health surveillance in high-risk occupational populations.

## Comparison with existing exposome-based brain aging models

Our framework complements rather than replaces existing exposome-based brain aging models. While exposome approaches broadly capture multiple environmental factors across the life course (63, 64), our model offers four distinctive features: (1) domain-specific focus on occupational dust with attention to particle physicochemical properties; (2) mechanistic integration of cellular senescence as a core pathway linking chronic exposure to neurovascular aging; (3) operationalization for occupational health as a closed-loop, actionable system (Figure 5); and (4) scalability through OCT-A screening and AI-enabled risk stratification. These features position our framework as a complementary, implementation-ready model for occupational populations, whereas exposome models provide broader but less domain-specific insights.

## Implementation feasibility: real-world considerations

The proposed multimodal MRI + OCT-A + AI pipeline faces substantial barriers to real-world implementation, particularly in low-resource occupational health settings.

High-resource settings: Implementation is feasible in academic centers and large occupational cohorts. Portable OCT-A devices enable screening at worksites (18); standardized MRI protocols exist for CSVD assessment (13); and open-source AI tools for WMH segmentation are increasingly available (56).

Low-resource settings: MRI remains cost-prohibitive. OCT-A offers a more accessible alternative due to lower cost, portability, and shorter acquisition time (55). However, OCT-A still requires trained personnel and equipment investment. The most realistic pathway is risk stratification: using OCT-A as a screening tool in high-exposure workers to identify candidates for MRI referral.

Practical barriers: Key obstacles include (1) equipment cost and availability; (2) lack of technical expertise for AI implementation; (3) fragmented occupational health records; (4) regulatory requirements for medical devices; and (5) unclear cost-effectiveness evidence.

Tiered implementation approach: We propose a tiered strategy based on resource availability: Tier 1 (high-resource): Full multimodal MRI + OCT-A + AI pipelines in centralized occupational health centers; Tier 2 (middle-resource): OCT-A screening with portable devices, combined with cognitive testing and traditional risk factor management; Tier 3 (low-resource): Enhanced health education, smoking cessation, hypertension control, and targeted screening of workers with longest exposure durations.

Future research should prioritize implementation science studies to evaluate feasibility, cost-effectiveness, and scalability of these approaches across diverse occupational settings.

## Conclusion

In conclusion, cross-sectional and limited longitudinal studies suggest an association between occupational dust exposure and CSVD imaging markers, including white matter hyperintensities, lacunar infarcts, and cerebral microbleeds. Several dose-response observations support a biological gradient, and experimental evidence provides plausible mechanisms involving systemic inflammation, endothelial dysfunction, and blood-brain barrier disruption. However, causal inference remains severely limited by the predominance of cross-sectional designs, potential biases (healthy worker survivor effect, exposure misclassification, residual confounding), and inconsistent adjustment for key confounders. Null findings exist in the literature and may be explained by methodological limitations rather than absence of true effects.

We propose an integrative framework (Figure 5) that links exposure science, mechanistic research, precision imaging, and AI-driven risk prediction. This framework is designed as a closed-loop, actionable system for occupational health surveillance, but its implementation requires prospective validation. Future efforts should prioritize: (1) prospective cohort studies with quantitative personal exposure monitoring, repeated neuroimaging, and rigorous confounder assessment; (2) validation of AI-enhanced risk stratification tools in multi-center settings; and (3) implementation research to evaluate the feasibility and cost-effectiveness of workplace-based brain health screening.

We advocate for cautious interpretation of current evidence and for inclusion of brain health considerations in occupational safety research agendas, while emphasizing that causality has not yet been established.
